# Green Synthesis of Titanium Dioxide Nanoparticles: Characterization and Evaluation of Their Potential for Photocatalytic and Dielectric Applications

**DOI:** 10.3390/molecules30244701

**Published:** 2025-12-08

**Authors:** Manal A. Awad, Khalid M. O. Ortashi, Wadha Alenazi, Fatimah S. Alfaifi, Asma A. Al-Huqail

**Affiliations:** 1 King Abdullah Institute for Nanotechnology, King Saud University, P.O. Box 2455, Riyadh 11451, Saudi Arabia; 2 Department of Chemical Engineering, King Saud University, P.O. Box 2455, Riyadh 11421, Saudi Arabia; ortashi9@ksu.edu.sa; 3 Department of Physics, College of Science, King Saud University, P.O. Box 2452, Riyadh 11459, Saudi Arabia; walenazi@ksu.edu.sa (W.A.); falfaifi@ksu.edu.sa (F.S.A.); 4 Chair of Climate Change, Environmental Development and Vegetation Cover, Department of Botany and Microbiology, College of Science, King Saud University, P.O. Box 2455, Riyadh 11451, Saudi Arabia

**Keywords:** green synthesis, anatase TiO_2_ NPs, photocatalytic efficiency, dielectric performance

## Abstract

This study investigated the dielectric and photocatalytic properties of green-synthesized titanium dioxide nanoparticles (TiO_2_ NPs), which are widely utilized semiconductor materials known for their excellent optical, structural, and electronic characteristics. The TiO_2_ NPs were synthesized via a green precipitation method from the aqueous extract of *Cymbopogon proximus*. A comprehensive set of analytical techniques—UV–Vis spectroscopy, XRD, FTIR, TEM, EDX, and DLS—was employed to determine their optical response, crystalline structure, functional groups, morphology, elemental composition, and particle size distribution. UV–Vis analysis revealed a characteristic absorption peak at 327 nm, and the band gap energy, calculated via the Tauc plot method, was 3.16 eV. The XRD results confirmed the formation of a tetragonal TiO_2_ phase with an average crystallite size of approximately 4 nm. TEM images further supported the spherical to quasitetragonal morphology and revealed that the aggregated clusters formed conjoint nanostructures. The photocatalytic activity of the TiO_2_ NPs was evaluated using a 0.5 mM RhB dye solution under UV–visible irradiation. The synthesized nanoparticles achieved a photodegradation efficiency of 97% after 50 h, with a corresponding rate constant of 0.073402 h^−1^, indicating their potential for effective photocatalytic pollutant removal. Furthermore, the dielectric behavior of the TiO_2_ NPs was examined at room temperature. The material exhibited a high dielectric constant at low frequencies due to interfacial (Maxwell–Wagner) polarization, along with frequency-dependent AC conductivity attributed to charge-carrier hopping mechanisms. These dielectric properties, combined with strong photocatalytic performance, underscore the suitability of green-synthesized TiO_2_ NPs for applications in environmental remediation, energy-storage devices, and advanced technologies.

## 1. Introduction

Nanotechnology focuses on engineering materials at the atomic and molecular scales, where a high surface-area-to-volume ratio imparts unique physicochemical properties. These nanoscale materials play vital roles in multiple sectors, including energy, electronics, imaging, environmental remediation, and molecular diagnostics [1]. NPs are broadly classified into organic systems (e.g., micelles and liposomes) and inorganic systems (e.g., metals, semiconductors, and magnetic nanostructures). Among inorganic nanomaterials, titanium dioxide (TiO_2_) is particularly prominent owing to its excellent thermal stability, optical activity, electronic response, and catalytic efficiency [2]. TiO_2_ has gained extensive industrial and scientific interest because of its desirable characteristics—nontoxicity, high chemical stability, strong oxidizing ability, and ease of synthesis [3]. These properties underpin its application in photocatalysis, where TiO_2_ functions as an efficient antiviral and antibacterial agent, facilitates the degradation of volatile organic compounds, promotes water splitting, and contributes to cancer cell destruction under irradiation [4]. TiO_2_ nanoparticles exist in three crystalline phases—anatase, rutile, and brookite—with anatase and rutile being the most technologically important owing to their structural stability and functional versatility [5,6]. Owing to their high refractive index, hydrophobicity, and excellent UV-blocking capability, TiO_2_ NPs are well suited for coatings, solar collectors, food additives, and water purification technologies.

In environmental and energy applications, TiO_2_ nanoparticles play essential roles in wastewater treatment, hydrogen production, and decomposition of hazardous contaminants. The effectiveness of these materials in antimicrobial systems, sensors, paints, and polymer composites further underscores their wide-ranging utility [7]. Despite their advantages, conventional physical and chemical synthesis routes often require high energy inputs and involve toxic reagents that generate hazardous byproducts [8]. These limitations have accelerated the shift toward greener, more sustainable synthesis approaches.

Green synthesis offers an environmentally benign alternative by employing plant extracts, microorganisms, or other biological systems as natural reducing and stabilizing agents [9]. Phytochemicals such as flavonoids, polyphenols, terpenoids, and alkaloids present in plant extracts play critical roles in controlling nanoparticle reduction, nucleation, and growth, ultimately influencing particle size, shape, and surface functionality [10]. Several plants have been successfully utilized for TiO_2_ NP synthesis, including *Azadirachta indica* (Neem) [11], *Camellia sinensis* (green tea) [12], *Aloe vera* [13], *Eucalyptus globulus* [14], *orange peel* [15], and *Daucus carota* (carrot) [16]. Green-synthesized TiO_2_ NPs exhibit strong photocatalytic performance under UV or visible light due to their ability to generate reactive oxygen species (ROS), enabling the breakdown of complex organic pollutants into nontoxic byproducts [17]. Additionally, TiO_2_ NPs demonstrate noteworthy dielectric behavior characterized by a high dielectric constant and low loss tangent, making them desirable in electronic devices such as capacitors, sensors, and energy-storage systems [18].

*Cymbopogon proximus* is a perennial aromatic grass belonging to the Poaceae family and is widely distributed in arid and semi-arid regions, including Sudan (locally known as Maharaib), North Africa, and Saudi Arabia (locally called Alazkher). According to previous literature, the plant is rich in essential oils and diverse secondary metabolites such as flavonoids, phenolic acids, terpenoids, and alkaloids [19,20,21]. These phytochemicals possess strong reducing and stabilizing capacities, making the plant extract suitable for green nanoparticle synthesis. Traditionally, *C. proximus* has been used for its diuretic, antispasmodic, and anti-inflammatory properties, and it is also valued in perfumery, cosmetics, and natural insect-repellent formulations [22,23]. In this study, *C. proximus* was selected as the green reducing and stabilizing agent because of its abundance, sustainability, and phytochemical richness. Its flavonoids, terpenoids, phenolics, and essential oils provide strong reducing and capping capabilities that support the eco-friendly formation of TiO_2_ nanoparticles with enhanced stability and functional performance [24,25,26]. Accordingly, this work investigated the green synthesis of TiO_2_ nanoparticles using *Cymbopogon proximus* extract [21], with a detailed assessment of their structural, optical, photocatalytic, and dielectric properties. By integrating sustainable synthesis with multifunctional performance, green-synthesized TiO_2_ NPs offer promising solutions to address technological and environmental challenges, aligning with the broader goals of sustainable nanotechnology.

## 2. Results and Discussion

**Figure 1:**
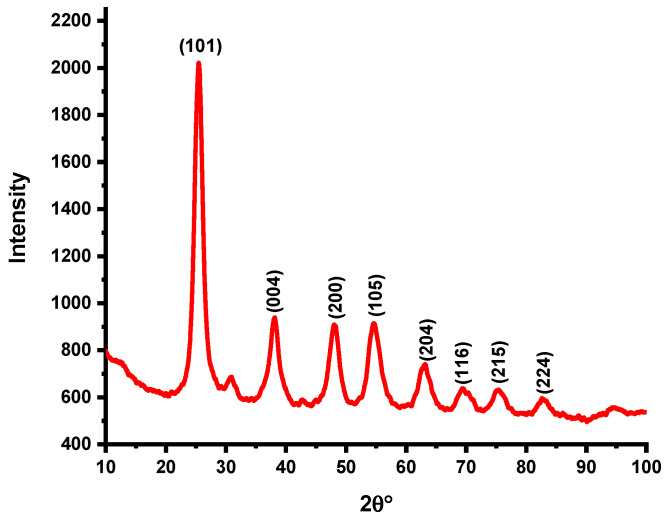
XRD analysis of synthesized TiO_2_ NPs.

Powder X-ray diffraction (XRD) analysis of the synthesized TiO_2_ nanoparticles was performed via a Bruker D8 ADVANCE diffractometer. The diffraction pattern confirmed that the TiO_2_ NPs crystallized in the highly pure tetragonal anatase phase, which is consistent with JCPDS card No. 21–1272. Characteristic diffraction peaks were observed at 25.44° (101), 38.13° (004), 48.02° (200), 54.49° (105), 62.87° (204), 69.71° (116), 75.25° (215), and 82.74° (224), as shown in Figure 1. The noticeable broadening of the main diffraction peak, along with the reduced relative peak intensity, indicates the formation of nanoparticles with very small crystallite sizes. This peak broadening is typical of nanostructured anatase TiO_2_ and is associated with an increased surface area, increased availability of active sites, and superior photocatalytic activity due to improved surface–reactant interactions [27]. Using the Debye–Scherrer equation and the most intense (101) diffraction peak, the average crystallite size (D) of the synthesized TiO_2_ nanoparticles was calculated to be approximately 4 nm, confirming the formation of ultrafine anatase nanocrystals.

**Figure 2:**
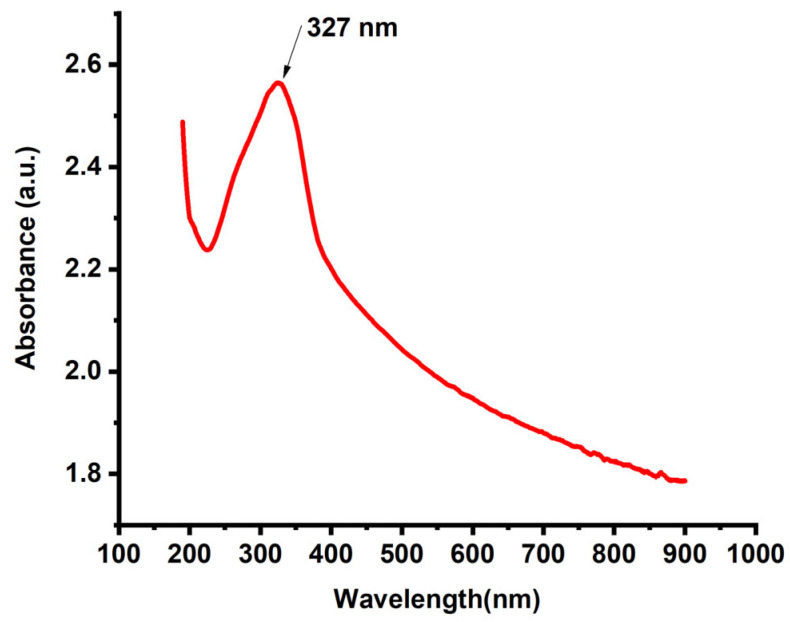
UV–visible absorption spectrum of anatase TiO_2_ NPs.

The optical properties of the synthesized TiO_2_ nanoparticles were examined via UV–Vis absorption spectroscopy, and the resulting spectrum is shown in Figure 2. The TiO_2_ NPs exhibited a strong and well-defined absorption peak at approximately 327 nm, which corresponds to the intrinsic band-to-band electronic transition characteristic of anatase TiO_2_. This prominent absorption feature confirms the successful formation of nanoscale TiO_2_, as TiO_2_ nanoparticles typically display absorption edges between 300 nm and 350 nm due to quantum size effects and surface-induced electronic transitions [28]. In addition to the main absorption peak, the gradual decrease in the absorbance extending toward the visible and near-infrared regions reflects the presence of tail states and possible surface defects, which are common in green-synthesized metal oxides due to the presence of phytochemical capping layers. The observed spectral characteristics confirm the successful synthesis of optically active, nanoscale TiO_2_ with a high degree of quantum confinement, making the material suitable for photocatalytic and optoelectronic applications.

**Figure 3:**
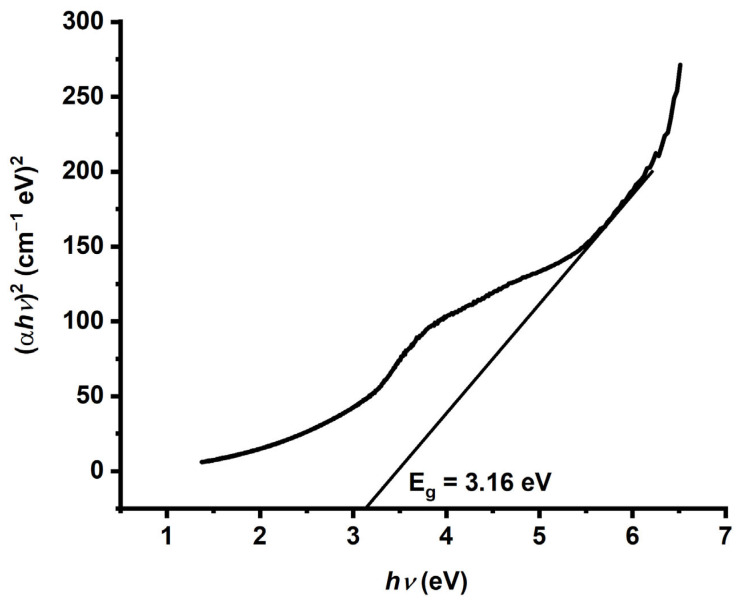
Bandgap energy (*hv)* curve of anatase TiO_2_ NPs.

The optical band gap of the synthesized TiO_2_ nanoparticles was evaluated via the Tauc plot method, which relates the absorption coefficient (α) to the photon energy (hν) for a direct allowed electronic transition. The Tauc plot (*α*ℎ*ν*)2 versus (*hν*) (Figure 3) exhibited a clear linear region, and extrapolation of this region to the energy axis gave an optical band gap of 3.16 eV. This bandgap value is slightly greater than that of bulk anatase TiO_2_ (≈3.0–3.2 eV), indicating that a modest blueshift is typically associated with a reduced crystallite size and quantum confinement effects. This behavior is consistent with the XRD findings, which revealed an average crystallite size of approximately 4 nm. The observed shift reflects alterations in the electronic structure and increased exciton binding energy, both of which enhance the photocatalytic capability of TiO_2_ NPs under UV illumination. The bandgap calculation, derived from the UV–Vis absorption spectrum via the Kubelka–Munk and Tauc models, confirms that the particle size and crystalline structure significantly influence the optical properties of TiO_2_ nanoparticles [29]. The measured value of 3.16 eV also corresponds to an absorption edge at ~327 nm, indicating that the material effectively absorbs UV light of shorter wavelengths, thereby generating electron–hole pairs capable of driving photocatalytic redox reactions [30].

**Figure 4:**
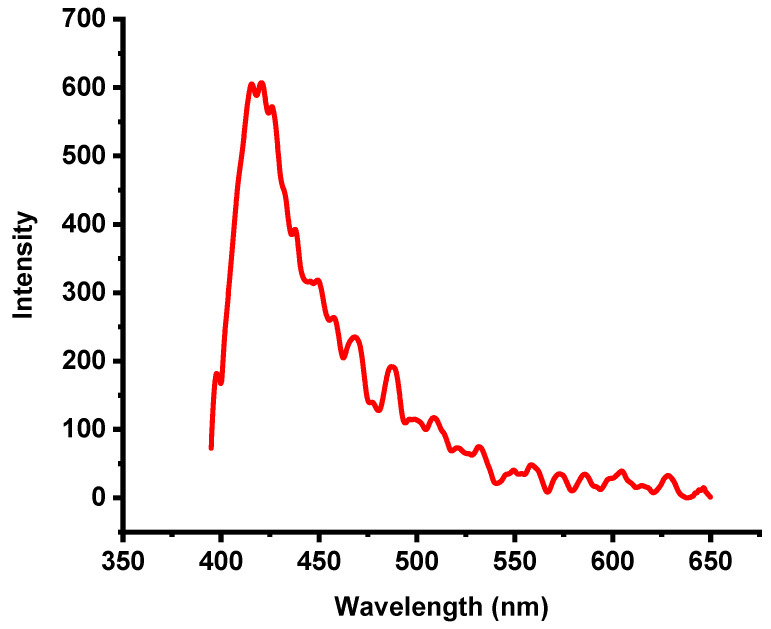
Photoluminescence and excitation spectrum of green anatase TiO_2_ NPs.

**Figure 5:**
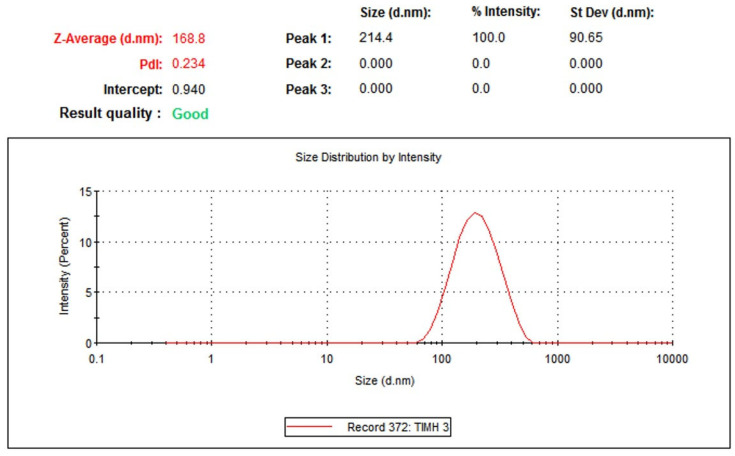
Average size of the green anatase TiO_2_ NPs measured by the DLS technique.

The excitation and photoluminescence (PL) spectra of the synthesized TiO_2_ nanoparticles were recorded at room temperature. Figure 4 shows the PL emission spectrum obtained upon excitation at 327 nm. The PL behavior of anatase-phase TiO_2_ is typically associated with three main physical processes: surface-state defects, self-trapped excitons, and oxygen vacancies [31]. Among these, the dominant surface defects generally arise from Ti^4+^ ions adjacent to oxygen-vacancy sites, which form during the nonequilibrium growth of nanoparticles. These defects play crucial roles in modifying the luminescence properties of TiO_2_, which is in agreement with earlier reports [32,33,34]. Upon excitation at 327 nm, electrons in TiO_2_ NPs are promoted from the valence band to the conduction band or to defect-related energy levels. The PL spectrum exhibits a visible emission peak at approximately 420 nm, originating from radiative recombination of charge carriers (Figure 5). This emission is attributed to electron transitions from shallow defect traps to free holes in the valence band. These defect states—often located within the bandgap—can arise from oxygen vacancies, surface imperfections due to the high surface-area-to-volume ratio of nanoparticles, or localized energy states introduced by excess titanium species [35].

Unlike intrinsic UV band-edge emission, the visible emission at 420 nm reflects defect-mediated recombination rather than direct band-to-band transitions, resulting in a redshifted PL response [36]. After excitation, electrons relax into lower-energy defect states before recombining, producing the observed longer-wavelength emission. The presence of these defect states significantly influences the optical and electronic behavior of TiO_2_ NPs. Notably, defects enhance visible-light responsiveness and can reduce electron–hole recombination by acting as trapping sites, thereby improving photocatalytic activity [37,38]. Rodríguez-Rojas et al. demonstrated that green synthesis routes can modulate the type and density of defect states, further optimizing TiO_2_ nanoparticles for pollutant-degradation applications [39].

The hydrodynamic diameter of the green-synthesized anatase TiO_2_ nanoparticles was measured via dynamic light scattering (DLS), as shown in Figure 5. DLS provides the effective particle size in suspension, which includes not only the nanoparticle core but also the surrounding hydration shell, stabilizing phytochemical layers and any weakly associated molecular species present in the colloidal medium [40]. The measured hydrodynamic diameter of 168.8 nm reflects the combined contribution of these factors, including naturally occurring nanoparticle aggregates that may form in solution [41]. This value is expected to be larger than the particle sizes observed through TEM, which determines the dry core size of individual nanoparticles without solvation or surface-bound biomolecules [42]. The polydispersity index (PDI) of the TiO_2_ nanoparticle suspension was 0.234, indicating a moderately narrow size distribution and good colloidal stability. A PDI value less than 0.3 is generally considered indicative of a uniform and stable nanoparticle system, whereas higher values suggest broader size distributions or aggregation tendencies [43]. The obtained PDI confirms that the green-synthesized anatase TiO_2_ NPs exhibit acceptable dispersion quality and remain relatively stable in aqueous suspensions.

**Figure 6:**
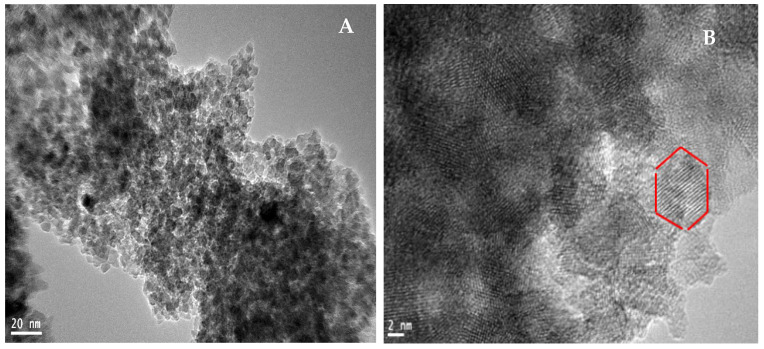
(**A**) TEM and (**B**) HRTEM images of green-synthesized TiO_2_ NPs, the red-highlighted region indicates the selected area for lattice-fringe analysis, confirming the crystalline structure and anatase phase of the TiO_2_ nanoparticles.

Transmission electron microscopy (TEM) was employed to investigate the morphology, size, and aggregation behavior of the synthesized TiO_2_ nanoparticles. As shown in Figure 6A,B, the TEM micrographs reveal conjoint spherical and clustered nanostructures, with the larger-scale image (20 nm scale bar) displaying aggregated spherical particles, whereas the higher-magnification image (2 nm scale bar) confirms the presence of well-defined spherical to quasitetragonal TiO_2_ nanocrystals. The surface characteristics of these nanomaterials are influenced by metal–oxygen and oxygen–hydroxyl (O–OH) bonding, which play critical roles in shaping spherical nanoparticles and promoting cluster formation [44,45]. According to Rathi et al. [46], phytochemicals present in plant extracts can regulate nanoparticle nucleation and growth, preventing excessive aggregation and influencing OH-related surface interactions. The observed aggregation in the TEM images may result from multiple factors, including incomplete surface passivation, particle–particle attractions in the absence of strong capping agents, and the intrinsically high surface energy of the nanoparticles, which promotes agglomeration to minimize the total free energy [47,48]. Despite these effects, the TEM results support the formation of crystalline TiO_2_ nanoparticles and are consistent with the XRD and UV–Vis findings.

**Figure 7:**
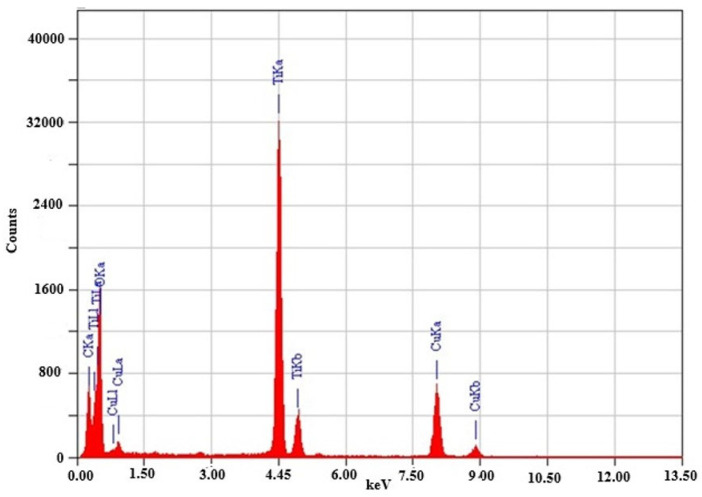
EDX analysis of the TiO_2_ nanoparticles.

The elemental composition was further confirmed by energy-dispersive X-ray (EDX) spectroscopy (Figure 7). The EDX spectrum shows distinct, high-intensity peaks corresponding to titanium and oxygen, confirming the successful synthesis of the TiO_2_ nanoparticles. Characteristic Ti peaks appear between 4.5 keV and 5.0 keV, with a prominent peak at approximately 4.5 keV, whereas oxygen peaks are present below 1 keV [49]. A copper (Cu) signal is also observed, originating from the copper TEM grid used during sample preparation. The elemental ratios indicate stoichiometric TiO_2_ with no detectable impurities, demonstrating the high purity of the synthesized nanoparticles. These results align well with previous literature reports on green-synthesized TiO_2_ nanomaterials [50,51]. Unlike metallic nanoparticles, which exhibit strong surface plasmon resonance, TiO_2_ nanoparticles primarily display optical absorption features arising from their electronic band structure. The absence of unwanted elemental contaminants and the confirmed stoichiometry highlight the purity and quality of the material, making the synthesized TiO_2_ NPs suitable for a range of applications, particularly photocatalysis, where composition, crystallinity, and purity are essential performance factors [52,53].

**Figure 8:**
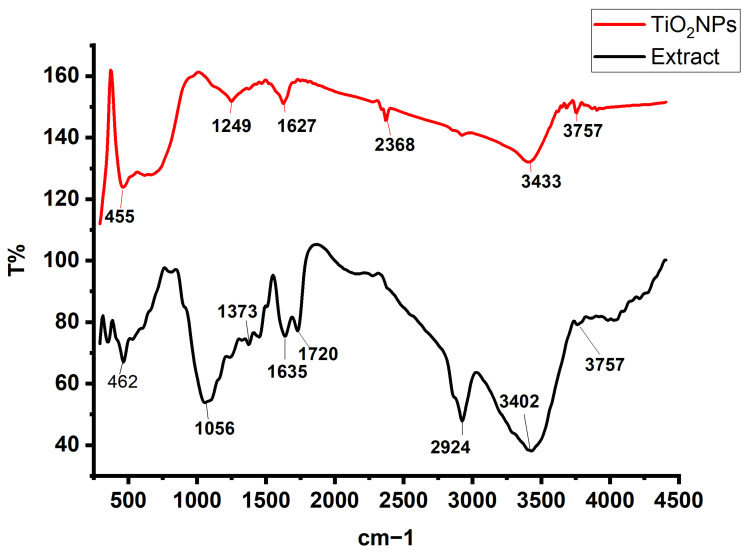
FTIR spectra of *C. proximus* extract and green-synthesized TiO_2_ NPs.

FTIR analysis was performed to confirm the functional groups, lattice vibrations, and structural integrity of the synthesized TiO_2_ nanoparticles. The resulting spectra (Figure 8) illustrate the characteristic vibrational modes of TiO_2_, as well as the organic and hydroxyl groups originating from the plant extract. These features collectively confirm the formation of anatase TiO_2_ and provide insights into surface chemistry modifications induced by green synthesis [54]. In the fingerprint region, distinct peaks at 462 cm^−1^, 617 cm^−1^, and 663 cm^−1^ were observed for the TiO_2_ nanoparticles. The band at 462 cm^−1^ corresponds to the Ti–O stretching vibration within the TiO_6_ octahedral units of anatase TiO_2_. The peak at 617 cm^−1^ is attributed to Ti–O–Ti bending modes, reflecting the connectivity of the octahedral framework. The appearance of a higher-energy Ti–O stretching band at 663 cm^−1^ is likely influenced by surface interactions, including the presence of phytochemical capping molecules or residual biomolecules from the plant extract. These interactions may slightly shift or broaden the characteristic Ti–O–Ti vibrations due to altered bond strengths or local surface environments [55,56]. Several additional peaks—1249 cm^−1^, 1627 cm^−1^, 2368 cm^−1^, 2924 cm^−1^, and 3757 cm^−1^—illustrate the contributions of organic and hydroxyl groups commonly associated with green-synthesized nanoparticles. The peak at 1249 cm^−1^ corresponds to C–O or C–O–C stretching vibrations from organic residues, such as esters or ethers, derived from the *C. proximus* extract. The band at 1627 cm^−1^ represents O–H bending vibrations from adsorbed water molecules or surface hydroxyl groups and may also include contributions from C=O stretching of plant-derived organic compounds [57]. The peak observed at 2368 cm^−1^ is associated with the asymmetric stretching of atmospheric CO_2_ adsorbed on the nanoparticle surface. The C–H stretching band at 2924 cm^−1^ indicates the presence of aliphatic groups from phytochemical stabilizers or organic residues [58]. The strong peak at 3757 cm^−1^ corresponds to free O–H stretching vibrations, confirming the presence of isolated surface hydroxyl groups on the TiO_2_ nanoparticles [59]. Together, these spectral features highlight the influence of green synthesis on the surface chemistry of TiO_2_ nanoparticles. Plant-derived biomolecules act as natural reducing and capping agents, introducing functional organic groups and enhancing the adsorption of hydroxyl species on the nanoparticle surface. These interactions not only confirm successful green synthesis but also improve surface reactivity and stability, which are advantageous for photocatalysis, adsorption, and other environmental applications [60].

**Figure 9:**
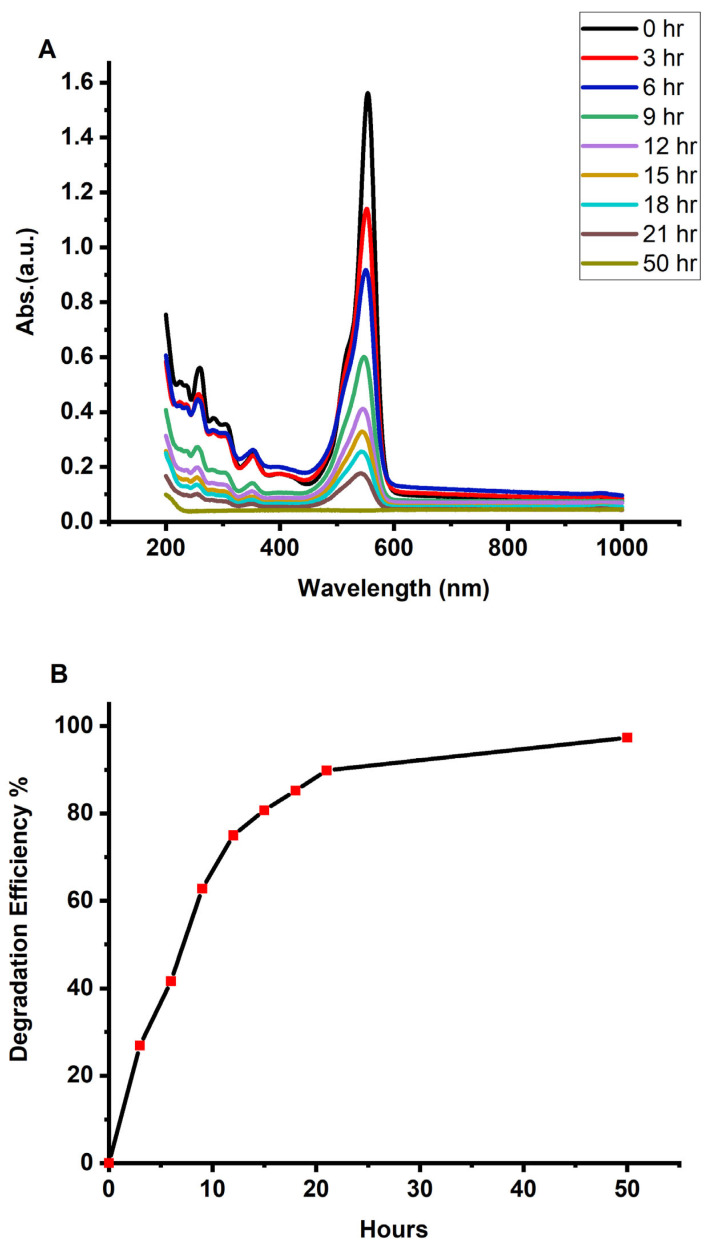
(**A**) Photocatalytic degradation of RhB dye in the presence of the synthesized TiO_2_ NP catalyst. (**B**) Degradation efficiency (%) of the resulting green TiO_2_ under UV irradiation.

The green-synthesized anatase TiO_2_ nanoparticles exhibited strong photocatalytic activity toward the degradation of Rhodamine B (RhB) dye. As shown in Figure 9A,B, the catalyst achieved 97% degradation after 50 h, demonstrating high photocatalytic efficiency and confirming its suitability for environmental remediation applications, such as wastewater treatment and organic pollutant removal. The substantial reduction in dye concentration indicates that the TiO_2_ NPs effectively utilized photogenerated charge carriers and reactive oxygen species to decompose the RhB molecules under UV irradiation. The degradation data were found to follow a pseudo–first-order kinetic model, yielding a rate constant of 0.073402 h^−1^, which reflects the reaction rate per unit time and highlights the effective interaction between RhB molecules and the TiO_2_ surface. Despite the relatively long total degradation time, the catalyst maintained continuous activity throughout the process, enabling nearly complete dye removal. The calculated half-life (t_1/2_) of 9.45 h confirms steady degradation behavior, indicating that the green-synthesized TiO_2_ NPs sustain efficient catalytic activity over the entire reaction period. The combination of high removal efficiency, a notable kinetic rate constant, and the ability to maintain photocatalytic performance over extended durations underscores the strong catalytic potential of the green-synthesized TiO_2_ nanoparticles. The use of *Cymbopogon proximus* extract in synthesis not only supports environmental sustainability but also produces nanoparticles with favorable surface chemistry and defect structures that enhance photocatalytic performance. Optimizing operational parameters—such as catalyst dosage, pH, and light intensity—may further reduce the reaction time while maintaining high degradation efficiency.

Table 1 compares the photocatalytic efficiency of the synthesized TiO_2_ NPs with values reported in the literature, demonstrating their competitive performance. This comparison provides deeper insight into the influence of different plant extracts, synthesis conditions, and reaction environments on photocatalytic outcomes, offering valuable information for future applications in hazardous dye degradation and water purification systems.

**Figure 10:**
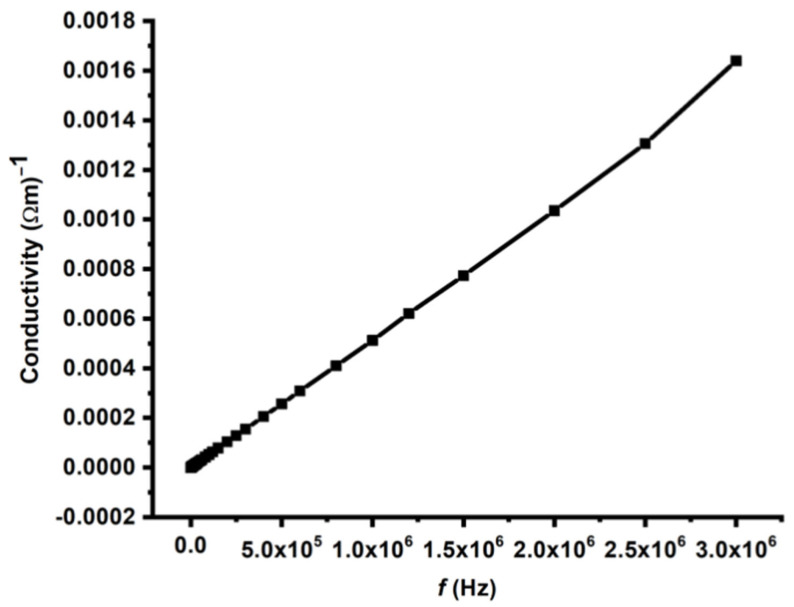
Frequency dependence of AC conductivity measured for the synthesized TiO_2_ NPs.

The dielectric behavior of the pelletized TiO_2_ nanoparticles was evaluated via the conventional parallel-plate capacitor technique. Dielectric properties are closely associated with the electro-optic response of a material and play a crucial role in microelectronics, where materials with controlled dielectric constants are essential for interlayer dielectric applications [72]. In this study, the frequency dependence of the dielectric constant (ε′) and AC conductivity (σ) of synthesized TiO_2_ nanoparticle pellets was measured at room temperature over a broad frequency range of 20 Hz–3 MHz. Figure 10 presents the AC conductivity profile of the TiO_2_ sample, which shows a gradual and nearly linear increase in conductivity with increasing frequency, particularly in the high-frequency region. This behavior is characteristic of many dielectric and nanostructured materials and is attributed to the frequency-dependent mobility of charge carriers within the material [73]. At lower frequencies, the conductivity remains relatively low because charge carriers are predominantly localized at grain boundaries, defect sites, and interfacial regions. These trapped charges are unable to respond effectively to slowly varying electric fields, resulting in limited conduction. As the frequency increases, the alternating electric field oscillates more rapidly, enabling localized charges to overcome potential barriers and participate in conduction. This phenomenon enhances the charge mobility and results in an increase in the AC conductivity. The nearly linear rise in conductivity at higher frequencies suggests the presence of a hopping conduction mechanism, where charge carriers hop between localized states such as defects or ions. The probability of such hops increases with frequency, which is consistent with the universal dielectric response model typically observed in heterogeneous or disordered systems, including nanomaterials [74]. The behavior observed here also supports the findings of Peláiz-Barranco and Dhaou, who reported that frequency-dependent conduction is strongly influenced by the microstructure of the material [75,76]. The synthesized TiO_2_ likely contains a complex network of interfaces, surface states, and defect centers that contribute to the charge transport dynamics. At higher frequencies, reduced interfacial polarization and enhanced charge-carrier mobility further contribute to the increase in conductivity. The frequency-dependent conductivity of the green-synthesized TiO_2_ nanoparticles highlights their potential for use in applications requiring controlled AC conduction, such as high-frequency electronic devices, capacitive components, and dielectric layers. These properties make the synthesized TiO_2_ NPs promising candidates for advanced microelectronic and energy-storage technologies where the dielectric and conduction behavior must be precisely tuned [77].

**Figure 11:**
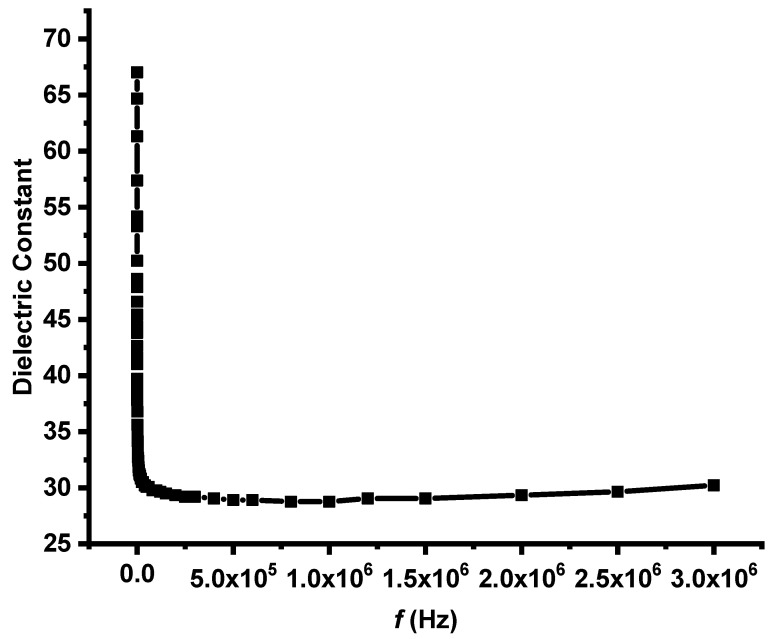
The frequency dependence of the dielectric constant measured for synthesized TiO_2_ NPs.

Figure 11 shows the frequency-dependent behavior of the dielectric constant (ε′) of the synthesized TiO_2_ nanoparticle sample. A clear dispersion pattern is observed: the dielectric constant is high at low frequencies and gradually decreases with increasing frequency, eventually reaching a nearly constant value in the high-frequency region. The dielectric response of a material reflects its polarization mechanisms and provides important insight into its electrical behavior. At low frequencies, TiO_2_ NPs display a large dielectric constant due to the dominance of interfacial or Maxwell–Wagner–Sillars (MWS) polarization. This mechanism arises from the accumulation of charge carriers at interfaces—such as grain boundaries, nanoparticle surfaces, and defect sites—where slow-moving ionic or defect-related species can follow the applied electric field. Their ability to align with the field results in enhanced polarization and, consequently, elevated ε′ values. As the frequency increases, the dielectric constant begins to decrease. This decline occurs because slower polarization mechanisms (interfacial and dipolar) can no longer respond to the rapidly oscillating electric field. At these intermediate to high frequencies, only faster polarization processes, such as electronic and atomic polarization, are able to keep pace. These intrinsic mechanisms involve rapid displacement of electrons or atomic positions and contribute less strongly to the overall dielectric response, resulting in lower ε′ values [78]. In the high-frequency region, the dielectric constant becomes almost frequency independent, indicating that only intrinsic polarization mechanisms are active. The stability of ε′ in this range reflects the fundamental dielectric nature of TiO_2_, which is influenced by its crystalline structure, bonding characteristics, and nanoparticle morphology. The observed behavior confirms that the synthesized TiO_2_ nanoparticles possess a heterogeneous and nanostructured architecture, where multiple polarization processes contribute to the overall dielectric response. These characteristics are typical of nanomaterials and are advantageous for a variety of electronic applications, including capacitors, dielectric resonators, and microelectronic components, where high ε′ at low frequencies and stable performance at high frequencies are desirable [79]. Furthermore, the elevated dielectric constant at low frequencies suggests the presence of defects, surface states, or trapped charges within the TiO_2_ nanoparticles, which enhance interfacial polarization. Conversely, the stable ε′ values at high frequencies emphasize the intrinsic dielectric quality of the anatase TiO_2_ phase, supporting its potential for high-performance dielectric and electronic applications [80].

## 3. Materials and Methods

### 3.1. Green Synthesis and Characterization of TiO_2_ NPs

**Figure 12:**
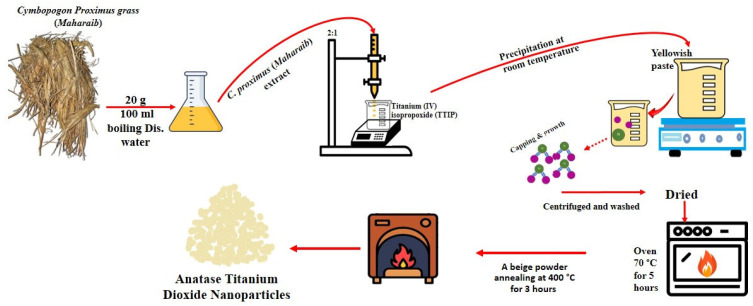
Schematic representation of the green synthesis process of TiO_2_ nanoparticles using *Cymbopogon proximus* extract.

The plant material was obtained from a local market in Riyadh, Saudi Arabia. Thirty grams of *C. proximus* (Alazkher or Maharaib grass) were thoroughly washed twice with tap water and once with distilled water. The cleaned material was air-dried under a hood for 24 h and then ground into a fine powder. Three grams of the powder were mixed with 100 mL of hot distilled water to prepare the plant extract, which was allowed to stand at room temperature overnight before being subjected to successive filtration steps. For nanoparticle synthesis, 20 mL of the filtered *C. proximus* extract was placed in a 100 mL glass beaker on a magnetic stirrer at room temperature. While stirring, 10 mL of titanium (IV) isopropoxide (TTIP) (Sigma-Aldrich, Stockholm, Sweden) was added dropwise to the extract. A yellow–yellowish paste gradually formed, and the reaction mixture exhibited a pH of approximately 6–7 during synthesis. After the addition, the mixture was stirred for an additional 3 h and then left undisturbed for 12 h. The resulting suspension was decanted to separate the sediment, which was then washed repeatedly with distilled water or ethanol and centrifuged three times at 14,000 rpm for 10 min to remove residual impurities. The collected precipitate was dried at 70 °C for 5 h to obtain a beige powder of TiO_2_ NPs, followed by calcination at 400 °C for 3 h at a heating rate of 20 °C/min (Figure 12) [21].

The synthesized TiO_2_ nanoparticles were characterized via a wide range of analytical techniques. Transmission electron microscopy (TEM; JEM-1400, JEOL, Tokyo, Japan) was employed to examine the particle size and morphology. UV–Vis absorption spectroscopy (Shimadzu UV-1800, Kyoto, Japan) was used to determine the optical properties, light absorption behavior, and bandgap energy of the TiO_2_ NPs across the 200–900 nm range. The elemental composition was analyzed via energy-dispersive X-ray spectroscopy (EDX) coupled with a JEM-2100F transmission electron microscope (JEOL Ltd., Tokyo, Japan). The hydrodynamic diameter and polydispersity index (PDI) of the colloidal nanoparticles were measured via dynamic light scattering (DLS) with a Zetasizer (ZEN3600, Malvern Nano Series, Malvern, UK). The surface functional groups present in both the nanoparticles and the plant extract were identified via Fourier transform infrared spectroscopy (FTIR; Shimadzu IR Prestige-21, Nakagyo-Ku, Japan) in the spectral range of 4000–400 cm^−1^. This analysis was carried out at the Chemistry Department, King Saud University, Female Students Campus, Riyadh, Saudi Arabia. The photoluminescence (PL) emission spectrum was recorded via a Fluorolog-3 spectrofluorometer (FL-3-11, Horiba Jobin Yvon, Irvine, CA, USA) to further assess the optical characteristics. The dielectric properties of the TiO_2_ nanoparticle pellets were evaluated in the frequency range of 2 Hz–3 MHz via a WAYNE KERR precision component analyzer (Model 6440B, Chichester, UK). Structural analysis was conducted via a Bruker D8 ADVANCE X-ray diffractometer (Bruker, Billerica, MA, USA) operated at 40 kV and 40 mA with Cu Kα radiation (λ = 1.5418 Å). The average crystallite size (D) of the TiO_2_ NPs was calculated via the Debye–Scherrer Equation (1):

(1) D= kʎβcosθ       

where D is the average crystallite size. K is the Scherrer constant (≈0.9). ʎ is the X-ray wavelength. β is the FWHM of the diffraction peak in radians. θ is the Bragg angle.

### 3.2. Photocatalytic Activity Study

The photocatalytic performance of the synthesized TiO_2_ nanoparticles was evaluated via the degradation of Rhodamine B (RhB, 0.5 mM) under both UV irradiation and direct solar light [24,25]. Twenty milliliters of the dye solution was transferred into a laboratory cuvette, followed by the addition of the TiO_2_ NP sample. The mixture was then exposed either to sunlight or to a controlled UV light source. UV–Vis absorption spectra were recorded at regular time intervals to monitor the degradation process, with the progressive decrease in the characteristic absorption peak of RhB used as an indicator of photocatalytic efficiency.

The degradation efficiency (DE%) was calculated via the following equation:

DE% = (A_0_ − A)/A_0_ × 100 (2)

where A_0_ is the initial absorption intensity, and A is the absorption intensity after photodegradation occurs.

Heterogeneous photocatalysis operates on the principle that a semiconductor can be activated by either artificial or natural light [25]. When photons strike a semiconductor with sufficient energy, electron–hole pairs are generated. In the case of TiO_2_ NPs, photons with energy equal to or greater than the band gap (λ ≈ 327 nm) excite electrons from the valence band (VB) to the conduction band (CB), producing electron–hole pairs as follows [26]:

(3) $$\mathrm{Green}\;\mathrm{Ti}O_{2}+\;hv(UV\;light)=\;{e^{-}}_{CB}+={h^{+}}_{VB}$$

The photogenerated electrons in the conduction band reduce dissolved oxygen molecules to form superoxide radicals ($•O_{2}^{-}$):

(4) $${e^{-}}_{CB\;}+\;O_{2}\;\to \;•O_{2}^{-}$$

Moreover, the photogenerated holes in the valence band oxidize water molecules to produce hydroxyl radicals (•OH):

(5) $${h^{+}}_{VB}+H_{2}O\to •OH$$

These reactive oxygen species (ROS), particularly superoxide ($•O_{2}^{-})$ and hydroxyl radicals (•OH), are highly potent oxidizing agents capable of attacking and degrading RhB molecules. The dye undergoes stepwise oxidative decomposition into smaller intermediates, which are eventually mineralized into carbon dioxide, water, and other nontoxic byproducts:

(6) $$\mathrm{RhB}\;\mathrm{dye}+•O_{2}^{-}/•OH\to \mathrm{Intermedates}\to CO_{2}+H_{2}O+\mathrm{Other products}$$

### 3.3. Dielectric Properties Evaluation

The dielectric behavior of the synthesized TiO_2_ nanoparticles was examined via a precision component analyzer (WAYNE KERR 6440B, Chichester, UK) over a frequency range of 100 Hz–3 MHz. Disc-shaped TiO_2_ pellets were positioned between a Cu–brass electrode assembly to measure their AC electrical response at room temperature. The system, which was operated through dedicated software integrated with a Keithley semiconductor characterization platform, allowed the determination of key dielectric and impedance parameters, including impedance (Z), capacitance (C), dielectric loss (tan δ), and related properties in both series and parallel modes.

The real part of the complex permittivity, known as the dielectric constant (ε′), was calculated from the measured capacitance via:

ε′ = Cd/(ε_0_ A) (7)

C is the measured capacitance of the sample.

d is the thickness of the sample.

A is the area of the electrode on the material (contact area).

*ε*_0_ is the permittivity of free space (8.854 × 10^−12^ F/m).

The imaginary part of the complex permittivity, known as the dielectric loss (ε″), which represents the energy dissipated as heat due to polarization relaxation, was calculated via:

ε″ = ε′ × tanδ(8)

where tanδ is the loss tangent or dissipation factor, measured directly by an impedance analyzer, and *ε*′ is the dielectric constant.

AC conductivity $\left(\alpha_{AC}\right)$ was determined from the dielectric parameters according to

(9) $$\alpha_{AC}=\varepsilon_{0}\varepsilon \prime \prime \;\;\omega \;tan\delta$$

where ω= 2πf is the angular frequency.

## 4. Conclusions

In conclusion, this study comprehensively examined the structural, optical, morphological, dielectric, and photocatalytic properties of green-synthesized TiO_2_ nanoparticles produced from *Cymbopogon proximus* (Alazkher) extract as a natural capping and stabilizing agent. The synthesized TiO_2_ NPs were extensively characterized via UV–Vis, PL, EDX, TEM, XRD, DLS, and FTIR analyses. XRD confirmed the formation of the anatase crystalline phase with an average crystallite size of approximately 4 nm. UV–Vis and Tauc analyses revealed a reduced optical band gap of 3.16 eV, indicative of quantum confinement effects in the nanosized TiO_2_. TEM images confirmed the formation of well-defined nanostructures, whereas FTIR confirmed characteristic Ti–O vibrations along with phytochemical functional groups derived from the plant extract. The photocatalytic performance of the TiO_2_ NPs, evaluated using a 0.5 mM Rhodamine B solution under UV irradiation, demonstrated a high degradation efficiency of 97% after 50 h, with a pseudo–first-order reaction rate constant of 0.073402 h^−1^. The progressive broadening and reduction of the absorbance peaks confirmed effective dye degradation and the active involvement of the nanoparticles in the photocatalytic process. Dielectric measurements further revealed excellent electrical behavior. The dielectric constant (ε′) was high at low frequencies due to interfacial (Maxwell–Wagner–Sillars) polarization and decreased with frequency as slower polarization mechanisms became ineffective. At higher frequencies, ε′ stabilized, reflecting the intrinsic dielectric nature of TiO_2_. The AC conductivity (σ_AC_) increased linearly with frequency, suggesting a hopping conduction mechanism and enhanced charge-carrier mobility, which are typical features of heterogeneous nanostructured materials. The combination of strong photocatalytic activity, nanoscale crystallinity, modified surface chemistry, and favorable dielectric properties demonstrated that the *C. proximus* extract serves as an effective, sustainable, and eco-friendly stabilizing and capping agent for the production of high-quality TiO_2_ nanomaterials. The green-synthesized TiO_2_ NPs exhibit excellent potential for environmental remediation, energy storage, and other advanced technological applications.

## Figures and Tables

**Table 1:** Plant-mediated Synthesis of TiO_2_ NPs for Pollutant-Degradation Applications.

*Plant Extract*	Dye Degraded	Degradation Percentage (%)	Irradiation Time	Irradiation Light	TiO_2_ NPs Dosage
*Echinops echinatus* [61]	Trypan blue	84%	120 min	Visible light irradiation	80 mg
*Jatropha curcas L.* [62] *Tragacanth gum*	Tannery wastewater	82.26% removal of chemical oxygen demand, and 76.48% removal of chromium	200 min	Sun light	5 g
*Syzygium cumini* [63]	Wastewater	75.5% removal in chemical oxygen demand (COD), and 82.53% removal in lead (Pb^2+^)	12 h	UV lamps of 15 W	0.3 g
*Impatiens rothii Hook.f. leaf* [64]	Methylene Blue (MB)	98%	100 min	150 W tungsten-halogen lamp	10–50 mg/L
*Justicia gendarussa* [65]	MB	Rate constant values were 0.999, 0.892 and 0.836 min^−1^	75 min	UV irradiation	10 mg/200 mL
*Polyvinyl Pyrrolidone* [66]	Methyl orange (MO)	94%	150 min	UV irradiation	0.1 g
*Nerium* *Oleander leaf* [67]	MB	98%	180 min	Solar irradiation	0.025 g
*Arabian coffee* *seeds husk* [68]	MB	98%	135 min	Solar irradiation	-
*Aloe vera* [69]	MB	99.6%	60 min	UV irradiation	90 mg/L
*Acorus calamus Leaf* [70]	RhB	96.59%	120 min	visible irradiation	25 mg/mL
*Moringa oleifera leaf* [71]	MB	99%	60 min	Solar light irradiation	50–100 mg
*Cymbopogon proximus—current study*	RhB	97% rate constant value was 0.073402 h^−1^	50 h	UV irradiation	1 mg/L

## Data Availability

The data supporting the findings of this study are available from the corresponding authors upon reasonable request.
